# Annual Report Veterinary Department

**Published:** 1883-01

**Authors:** 


ANNUAL REPORT OF THE VETERINARY DEPARTMENT OF THE PRIVY COUNCIL OFFICE FOR THE YEAR 1881, WITH AN APPENDIX. 8vo., 275 pages. London, 1882.

Prof. G. T. Brown sends us a copy of the above mentioned report which is replete with valuable information. The appendix consists of an elaborate report on the diseases which have existed among animals of Great Britain and foreign countries in 1881, together with statistical tables showing the number of animals imported in the various parts of the kingdom, etc.

